# A sense of security in palliative homecare in a Norwegian municipality; dyadic comparisons of the perceptions of patients and relatives - a quantitative study

**DOI:** 10.1186/s12904-020-0513-7

**Published:** 2020-01-11

**Authors:** Reidun Hov, Bente Bjørsland, Bente Ødegård Kjøs, Bodil Wilde-Larsson

**Affiliations:** 1 Department of Nursing, Inland Norway University of Applied Sciences, Elverum, Norway; 2 Centre for Development of Institutional and Home Care Services, Hamar Municipality, Hedmark Norway; 3 Department of Health Science, Discipline of Nursing Science, Karlstad University, Karlstad, Sweden

**Keywords:** Security, Quality of palliative homecare, Patient and relative dyads, Quantitative

## Abstract

**Background:**

As palliative care increasingly takes place in patients’ homes, perceptions of security among patients in the late palliative phase and their relatives are important.

**Aim:**

To describe and compare patient-relative dyads regarding their perceptions of security in palliative homecare, including the perceived security of the actual care given to the patients, as well as the subjective importance of that care.

**Methods:**

A cross sectional questionnaire study including 32 patient-relative dyads was conducted in an urban municipality in Norway. Patients were in a late palliative phase and received palliative homecare. Each patient proposed one relative. Data were collected using a modified version of the Quality from the Patients’ Perspective instrument (QPP), which focuses on security and comprises three dimensions: medical-technical competence, identity-orientation approach and physical-technical conditions. Context-specific scales containing four aspects (competence, continuity, coordination/cooperation, availability) were added. The instrument contains two response scales; perceived reality (PR) and subjective importance (SI). Data were analysed by descriptive statistics, Chi-squared test, T-test and Wilcoxon’s signed rank test.

**Results:**

Patients had high mean scores on the PR-scale for the sense of security in palliative homecare in the dimensions of medical-technical competence and physical-technical conditions. There were three low mean scores on the PR-scale: the aspect of continuity from patients and the aspects of continuity and coordination/cooperation from relatives.

The patients scored the SI scale statistically significantly higher than the PR scale in the identity-orientation approach dimension and in the aspect of continuity, while relatives did so in all dimensions and aspects.

The intra-dyadic patient-relative comparisons show statistically significant lower scores from relatives on the PR-scale in the dimensions of medical-technical competence, physical-technical conditions, identity-orientation approach and the aspect coordination/cooperation.

**Conclusions:**

There are several statistically significant differences between patients and relatives’ perceptions of security in the palliative homecare received (PR) compared with the subjective importance of the care (SI) and statistically significant differences in the patient-relative dyads in PR. A relatively mutual sense of security in palliative homecare is important for patient-relative dyads, as relatives often provide care and act as patients’ spokespersons. What they assess as important can guide the development of palliative homecare.

## Background

The World Health Organisation (WHO) has stated that quality care is provided when the preferences of patients and relatives are taken into account and that their perceptions of care constitute dimensions of quality care [1]. Quality palliative care is important for meeting the needs of patients in a palliative phase of their illness [2], as they often suffer pain and other symptoms, loss of bodily and psychological functions and social relationships [3].

The World Health Assembly has indicated that health systems have an ethical responsibility to provide palliative care [4], for instance to meet the preferences of patients who want to stay in their own homes for as long as possible [5, 6] or die there [7, 8]. This is in line with the statement of the WHO that palliative care should be integrated into primary health care in the municipalities [9]. In Norway, where the present study was conducted, the policy in recent years has been to replace hospital-based care and treatment with community health care in or close to patients’ homes [10, 11].

A relative is often the key to meeting the patients’ preferences as well as achieving political aims [12]. Many relatives take on the responsibility for providing practical and medical care, in addition to social and emotional support, while at the same time facing the threat of losing a loved one [13]. The consequences can be contradictory: a perception of performing a meaningful mission and a feeling of reward by contributing to the fulfilment of the patient’s preferences, while at the same time physical and psychological stress and health problems may occur [14–16]. The above-mentioned concurrent preferences of patients and relatives as well as the intentions of society imply the need to focus on quality in palliative homecare.

A sense of security is reported to be an aspect of quality care [17] and a key component of palliative homecare [18–21]. Studies have revealed that patients and relatives’ perceptions of security in homecare were fairly similar and encompassed a trusting relationship with the nurse, feeling respected, recognized, informed and being involved in the treatment and care [19, 21]. It was also important to live as normal an everyday life as possible and that the healthcare professionals were reliable, competent and available when needed. Studies have demonstrated the significance of continuity [22, 23] and timely access to the required care, treatment and services [20, 24]. In addition, relatives have highlighted the importance of obtaining respite [21].

Corresponding perceptions of palliative homecare from the patient-relative dyad may be of importance, as relatives often assume the role of informal caregivers and patient spokespersons [15, 25]. However, a review reported that although relatives had less knowledge of subjective aspects of the patients’ experiences, they were aware of the quality of care and several symptoms [26]. A study about the quality of palliative homecare as perceived by dyads of patients with lung cancer-relatives shows high agreement in the ratings of the quality of the care received, but several differences in their perceptions of the importance of quality care [27]. Another study about perceptions of quality in advanced homecare from patient-relative dyads found a high degree of congruence between relatives’ opinions of how the patient perceived the care [28].

Although a sense of security has been found to be important for patients and relatives in palliative homecare, there are few studies about perceptions of security and the importance of such care from the dyadic perspectives of patients-relatives.

The aim was to describe and compare patient-relative dyads regarding their perceptions of security in palliative homecare, including the perceived security of the actual care given to the patients, as well as the subjective importance of that care.

## Methods

### Design

This is a cross sectional questionnaire study.

### Setting

In Norway, a municipality is a council area that can be a city, town or other district, possessing corporate existence and its own local government. The Norwegian municipalities (*n* = 422) [29] are responsible for providing homecare services for the population (*n* = 5.3 million) [30]. Homecare services include homecare nursing and practical assistance to meet the needs of each patient [31]. Palliative care is organised in specialised and non-specialised services, where mainly the latter serve patients in the municipalities [32]. This means that the healthcare professionals care for all patients who need homecare, including those who require palliative care.

The participants in this study were recruited from an urban municipality with about 30,000 inhabitants in the southeast of Norway. The homecare is organized in self-directed work teams (SDWT) on day and evening shifts. The basic idea behind a SDWT is that the team members are empowered and organize the service to meet the needs of the patients and their relatives, while ensuring optimal utilization of resources, continuity of care and appropriate competence [33–35]. A SDWT consists of a small group of healthcare professionals, including one who has the role of primary contact, which means that she/he provides daily care to “her/his” patient, including planning, evaluation and documentation.

There are three palliative SDWTs in the municipality, each of which cares for an average of 28 patients. They not only cover patients in the early or late palliative phase or terminal phase, but also seriously ill patients who are receiving curative treatment. A SDWT is made up of five registered nurses (RNs), of whom at least one has a specialist education in oncology nursing, two enrolled nurses and one assistant without any health education. A registered nurse (RN) who is responsible for organising the daily care leads each SDWT. The palliative SDWTs are non-specialised because they are not fully staffed by healthcare professionals, thus do not provide a multidisciplinary palliative approach. The patient’s general practitioner or physicians at the hospital deal with medical aspects during the day. In the evening, at night, on weekends and holidays this becomes the responsibility of physicians at the emergency department.

Two RNs specialised in oncology nursing work across the SDWTs to support seriously ill and dying patients and their family members as well as supervising the SDWT members. One of these nurses is entitled *cancer care coordinator* (100% position) and the other *cancer nurse* (50% position).

### Participants

This article is part of a study of 60 patients receiving palliative homecare in Norway and 38 of their relatives.

The present paper includes 32 patient-relative dyads. A patient-relative dyad refers to the patient and the person she/he has named as her/his closest relative. The inclusion criteria for patients and relatives were: ^1)^ ≥ 18 years, ^2)^ documentation showing that the patient was in a late palliative phase (with an advanced malignant or non-malignant illness) and that patient and relative were informed about the situation, ^3)^ ability to understand spoken Norwegian and ^4)^ competent to make decisions (in the opinion of the SDWT leader). Moreover, the patients should ^5)^ receive help from the cancer care coordinator or the palliative SDWT and ^6)^ the healthcare professionals should answer “no” to the 1 year life expectancy question: “Would you be surprised if this patient dies within the next year”? [36]. Relatives were included when ^7)^ proposed by the patient, ^8)^ the patient had given her/his consent for the relative to participate and ^9)^ the relative should be able to read and write Norwegian.

It was difficult to estimate the eligible sample, but the cancer care coordinator who keeps track of patients in the palliative phase in all SDWTs reckoned about 180 eligible patients. The patients were consecutively sampled by the SDWT leaders. About 95 were excluded due to not meeting inclusion criteria 2, 3, 4, 6 and because the SDWT leaders reported a heavy workload and thus forgot to invite patients to participate. Of the 85 patients who were asked to participate, four refused immediately, 16 failed to return the written consent and five died before the interview took place. Forty-three patients gave their consent to ask their relatives to participate and 38 relatives returned the questionnaire. Finally, the participants constituted 32 patient-relative dyads.

### The questionnaire

#### Participants’ characteristics

Characteristics of patients-relatives (29/28 items) consisted of age, gender, language/ethnicity, education, diagnosis, living conditions, support from the healthcare system, family and friends and preferred location of care. Relatives were also asked about their need for respite.

One question (EQ VAS) from the EQ-5D-5 L questionnaire [37, 38] measured the respondent’s self-rated health on a vertical visual analogue scale with endpoints from “Best imaginable health” (100), to “Worst imaginable health” (0). An overview of the demographic and health-related characteristics of the patient-relative dyads is presented in Table 1.

**Table 1:** Characteristics of patients and relatives dyads in palliative home care (*n* = 32)

	Patients	Relatives	*P*
Gender n (%)			
Women	19 (59%)	21 (66%)	
Men	13 (41%)	11 (34%)	
Age, mean score (SD)	73,75 (11.77)	59,56 (13.66)	< 0,001
Range	51–95	30–89	
Educational level n(%)			0.027
< Secondary school	9 (28%)	7 (22%)	
Secondary school	12 (37%)	15 (47%)	
University/U.College	6 (19%)	10 (31%)	
Other	4 (13%)		
Missing	1 (3%)		
Occupational status n(%)			
Employed		21 (66%)	
Retired	22 (69%)	8 (25%)	
On sick leave/disabled	10 (31%)	2 (6%)	
Other		1 (3%)	
Contact with family and friends n(%)			
Sufficient	28 (88%)	24 (75%)	
Want more contact	2 (6%)	7 (22%)	
Want less contact Missing	2 (6%)	1 (3%)	
The patient receives enough help n(%)			
Yes	28 (88%)	30 (94%)	
No	1 (3%)	2 (6%)	
Missing	3 (9%)		
Primary contact in home care n(%)			
Yes	17 (53%)	20 (63%)	
No	14 (44%)	10 (31%)	
Missing	1 (3%)	2 (6%)	
Know how to obtain help if something unanticipated occurs n(%)			
Yes	23 (72%)	28 (87%)	
No	6 (19%)	4 (13%)	
Missing	3 (9%)		
The wanted location of care in the future n(%)			
At home	29 (91%)	15 (47%)	< 0.001
At home combined with institution	1 (3%)	15 (47%)	
Institution Missing	2 (6%)	2 (6%)	
Self-rated Health VAS-scale (EQ-5DL) mean/range (SD)	52.42/10–90 (20.55)	79.17/35–100 (14.72)	< 0.001

Age and self-rated health of patients and relatives analysed by the T-test

Gender, educational level, preferred care location analysed by the Chi-squared test (X^2^)

The following information about patients and relatives is not shown in the table. All but one patient and all relatives were ethnic Norwegians. Furthermore, 13 of the patients lived alone, 19 had more than one diagnosis, 17 suffered from cancer, 16 had received palliative homecare for more than 6 months several times a day (*n* = 11) or daily (*n* = 6) from RNs (*n* = 26), enrolled nurses (*n* = 20) and/or the cancer care coordinator/cancer nurse (*n* = 15). In the 2 to 3 weeks prior to the interview, 12 patients had received treatment from a general practitioner, 20 had been hospitalized/received polyclinic treatment and 10 spent one or more days each week at a day hospice. Of the relatives, 24 cared for the patient part-time and 17 had no respite from this responsibility.

#### Quality from the patient’s perspective instrument – a modified version

Perceptions of security in palliative homecare were measured by a modified version of the Quality from the Patient’s Perspective (QPP) instrument. This patient-centred instrument was developed from a theoretical model of quality of care and research about patients’ perceptions of care quality [39, 40]. The theoretical framework includes four quality of care dimensions: the medical-technical competence of the caregiver (MT), the physical-technical conditions of the care organisation (PT), identity-orientation in caregivers’ attitudes and actions (ID) and the socio-cultural atmosphere of the care organisation (SC) (56 items full version) [39, 41]. The QPP has been found to be of a high standard [42], with robust psychometric characteristics in different languages and settings [41, 43–45].

A modified version of the QPP was developed in this study, based on results from research about security in palliative homecare [18, 21] and the researchers’ knowledge of the field. It consists of 44 items, of which 26 were chosen from the original QPP: MT (7), PT (1) and ID (18). Eighteen new items were added as context-specific security-related scales representing the following four aspects: competence (4 items), continuity (4 items), coordination/cooperation (4 items) and availability (6 items). Theoretical support for the chosen four security-related aspects and their content was found in all dimensions and their respective items as shown in Appendix.

Each item consists of a statement about the perception of security in palliative homecare in terms of the perceived reality (PR): “This is what I experience/what I think the patient experiences” and the subjective importance (SI): “This is how important it is to me/how important I think it is for the patient”. The statements were rated on a four-point Likert type scale. For the PR statement the scale was 1 (totally insecure), 2 (fairly insecure), 3 (fairly secure), 4 (totally secure) and for the SI statement the scale was 1 (of no importance), 2 (of some importance), 3 (of great importance), 4 (of very great importance). A non-applicable response alternative was available for both statements.

In a more formal sense, with the exception of Cronbach alpha calculations, no systematic test of the psychometric properties of the modified version has so far been performed because of the burdensome work involved in collecting additional data. This obviously also applies to the modified version of the QPP in the present study and the four newly constructed context-specific security-related scales. However, this study provides a detailed presentation of reliability coefficients (Cronbach’s alpha, see Table 2). In addition, visual inspection-based comparisons have been made with results obtained in a previous study from Norway using the modified QPP [46]. Fairly similar results were obtained, although the mean scores reported in the present study are slightly higher.

**Table 2:** Reliability test (Cronbach’s α) of the modified sense of security QPP scale in palliative home care

	Patients		Relatives	
	Perceived reality	Subjective importance	Perceived reality	Subjective importance
Modified QPP				
Dimensions				
Medical-technical competence (MT) (7 items)	0.86	0.74	0.82	0.70
Physical-technical competence (PT) (1 item)	–	–	–	–
Identity-oriented approach (ID) (18 items)	0.82	0.70	0.91	0.92
Context-specific scales				
Aspects				
Competence (4 items)	0.64	0.74	0.89	0.91
Continuity (4 items)	0.73	0.74	0.79	0.84
Coordination/collaboration (4 items)	0.62	0.87	0.91	0.82
Availability (6 items)	0.75	0.57	0.84	0.89

### Data collection

A pilot study was conducted in October 2013 including a dyad of five patients in a palliative phase/five relatives recruited from one SDWT. The participants answered the questionnaire and provided feedback on its length, relevance, complexity and the instructions about filling it in. The data from the pilot study were included in the total material as the feedback only concerned minor changes to the wording. The data collection continued until July 2015.

Two researchers (RH or BB) interviewed all patients in their homes by asking the questions in the questionnaire and filling it in. The response scales were enlarged on a separate sheet to make answering easier. The interviews lasted from 30 to 90 min. Some of the longest interviews included pauses because the patient felt tired.

The researchers (RH or BB) distributed the questionnaire and written information to the respective relatives. The relatives filled in the questionnaire by themselves.

All questionnaires were coded with numbers where the patient-relative formed a matching pair.

### Ethics

Ethical approval adhered to Norwegian regulations and law. The Regional Committee for Medical and Health Research Ethics in South East Norway assessed the study and stated that it did not require their approval (Reg.no 2013/679 A). Therefore, the Data Protection Authority of the Norwegian Centre for Research Data (NSD) approved the study (Reg.no. 34536). The head of the healthcare services in the municipality gave local approval for the study.

All patients provided their informed written consent. They were also asked for permission to request their relatives to participate. If they consented, the relatives received written information and completing the questionnaire was deemed informed consent. Patients and relatives were informed about the integrity, voluntariness and confidentiality of the study and assured that personal identification would be impossible in the published results [47].

Patients and relatives received the opportunity for extra support after the interview from one of the SDWT nurses.

The EuroQol Group gave permission to use the EQ-5D-5 L questionnaires.

### Data analysis

Data were analysed using IBM SPSS Statistics, version 24. Characteristics of patients and relatives and self-rated health were analysed by means of descriptive statistics. Potential differences between the age and educational level of the patients and relatives were analysed by the T-test, while gender and the preferred care location were analysed by the Chi-squared test (X^2^). The Wilcoxon’s signed rank test was used to examine the potential differences in perceptions of security between PR and SI for patients and relatives and the patient-relative dyads. The mean value in each dimension and aspect was calculated by adding the item scores and dividing by the number of items answered.

The Cronbach’s α coefficient was used to test internal consistency except for the PT dimension as it only contained one item. The Cronbach’s α coefficient is given for each dimension and aspect (Table 2) and ≥ 0.7 was regarded as desirable [48]. The Cronbach’s α coefficient ranged from 0.57 to 0.92, with < 70 for the context-specific scale, aspect competence (α = 0.64, patient PR), coordination/cooperation (α = 0.62, patient PR) and availability (α = 0.57, patient SI).

## Results

### Patients and relatives’ perceptions of security in palliative homecare

The results of the modified version of the QPP are presented in Table 3 as mean values, *p* and *z* values on dimensions and context-specific scales as aspects of PR and SI.

**Table 3:** Dyads of patients’ and relatives’ perceptions of security in palliative home care nursing; perceived reality and subjective importance by dimensions and context-specific scales

	Perceived reality		*Z*	*P*	Subjective importance		*Z*	*P*
	Patients	Relatives			Patients	Relatives		
	Mean (SD)	Mean (SD)			Mean(SD)	Mean (SD)		
Dimensions								
Medical-technical competence	3.57 (.49)	3.05 (.43)	2.023	.043	3.94 (.08)	3.78 (.28)	1.000	.317
Physical – technical competence	3.74 (.44)	3.46 (.50)		.034	3.74 (.44)	3.76 (.43)		
Identity-oriented approach	3.41 (.37)	3.29 (.38)	2.106	.035	3.49 (.26)	3.60 (.38)	1.687	.092
Context-specific scales								
Aspects								
Competence (4)	3.50 (.46)	3.27 (.52)	1.279	.201	3.69 (.38)	3.81 (.35)	0.917	.359
Continuity (4)	2.87 (.71)	2.59 (.71)	1.340	.180	3.26 (.63)	3.43 (.60)	0.885	.376
Coordination and cooperation (4)	3.40 (.60)	2.95 (.64)	2.034	.042	3.61 (.47)	3.71 (.39)	0.879	.380
Availability (6)	3.36 (.47)	3.22 (.31)	1.310	.258	3.54 (.34)	3.72 (.36)	0.632	.527

Scale Perceived reality: 1 = Totally insecure, 2 = Fairly insecure, 3 = Fairly secure, 4 = Totally secure

Scale Subjective importance: 1 = Of no importance, 2 = Of some importance, 3 = Of great importance, 4 = Of very great importance

Z scores of 1.96 (or higher) indicate a 95% confidence interval for a two tailed test and directionality (Wilcoxon’s signed ranked test)

*P*-values refer to the testing of difference between paired- sample (within patient-relative dyads) (Wilcoxon signed rank test)

In accordance with another study [46], mean scores of ≥3.55 are valued high and < 3.0 low.

The dimensions and context-specific scales show both high and low mean scores from the group of patients and no high mean scores from the group of relatives concerning the sense of security in the palliative homecare received (PR) (Table 3). The high mean scores from patients are on the dimensions MT (3.57) and PT (3.74). The low mean scores from patients are on the context-specific scales in the aspect of continuity (2.87), while from relatives they are in the aspects of continuity (2.59) and coordination/cooperation (2.95).

The patients’ mean scores are statistically significantly higher on one (ID) of three dimensions and one (continuity) of four aspects on the SI-scale compared to the PR-scale. The relatives’ mean scores are statistically significantly higher for SI than for PR on all dimensions and in all aspects on the context-specific scales.

### Patient-relative dyads’ perceptions of security in palliative homecare

The intra-dyadic comparisons between patients and relatives show statistically significant lower scores from relatives than patients on the PR-scale on the dimensions MT, PT and ID and the aspect coordination/cooperation on the context-specific scales. The mean values on the SI-scale show no statistically significant differences between patients and relatives. However, the mean value scores of patients are higher than those of relatives in one dimension (MT), while relatives had higher mean scores in all other dimensions as well as in all aspects on the context specific scales.

## Discussion

In palliative care, patients and relatives are viewed as a unit with the aim of enhancing their quality of life and well-being [49]. This study from a Norwegian municipality describes the sense of security in palliative homecare, including the care received (PR) and the subjective importance of that care (SI) as perceived by groups of patients and relatives and patient-relative dyads.

Thirteen of the 32 patients in the present study lived alone. Despite the fact that the number of participants is low, it is worth mentioning that many patients live alone, even in the palliative phase [12, 50, 51]. We need to be aware that changes in family structures from extended to nuclear families in western society will lead to more people, especially older ones, living alone. In Europe [52], including Scandinavia [53, 54], loneliness is a growing health problem. Patients in the late palliative phase often require comprehensive assistance due to their complex needs, and their relatives are referred to as the glue in the patients’ palliative homecare [13]. Even if patients have family members who want to help, they may be busy with other obligations such as work, home and children [55]. It can therefore be challenging for patients to remain at home for as long as they would like. It has been found that older patients with cancer who live alone make plans for death, but not for dying [56]. This underlines the need for healthcare professionals to focus on advanced care planning, especially for patients living alone, taking account of the fact that care aid support and health technology can have the potential to provide them with a sense of security [57, 58]. Furthermore, cohabitation of patients/relatives has been shown to either affect agreement about the perceptions of the quality of care positively [28] or not at all [27]. More studies are needed about what and how demographic aspects influence patients/relatives’ perceptions of security in palliative homecare.

The present study shows differences within patient-relative dyads when assessing the sense of security in the palliative care received, with significantly lower scores from relatives in all three dimensions (Medical-Technical competence (MT), Physical-Technical conditions (PT), Identity-Orientation (ID)) and in one aspect (coordination/cooperation).

The MT dimension is about medical treatment and care, a field that is often complex for patients in the late palliative phase [3]. Effective pain and symptom control for these patients is of the utmost importance and both patients and relatives need to be able to rely on the high quality of palliative care [59]. However, studies have shown that older patients with cancer have lower priorities for palliative care than younger ones [60] and that medication errors are common in homecare and can lead to patient harm [20, 61]. There is a great deal of knowledge available about these issues and we consider that it is time to ensure competent help with pain and symptoms, regardless of the age and location of patients in the palliative phase. If relatives perceive inadequate treatment and care, they may have to fill the gap. A lower sense of security may put them at greater risk of illness because of anxiety [25, 62], physical and psychological distress [63] and fear of being unable to cope [5, 24, 59]. Even 1 year after a patient’s death, relatives’ well-being has been shown to be predicted by their sense of security during the palliative care [64]. A sense of security in palliative homecare is therefore important for ensuring the achievement of political goals to prevent illness in relatives and fulfil the wishes of those patients who want to remain at home for as long as possible and/or die there [65]. It is necessary for healthcare professionals to routinely clarify relatives’ sense of security in the care and treatment as well as identify and meet their support needs.

The ID dimension concerns communication, respect, empathy, information and involvement. The latter is fundamental in modern health services, as well as for patients in the palliative phase [66] and their relatives [67]. It is important that relatives are not made to feel like spectators in the patient’s palliative treatment and care. Being involved in decision-making can improve relatives’ satisfaction with the quality of care [68], increase the possibilities of receiving care tailored to the patient’s specific needs and preferences and adapted to family life [16, 69]. Even if healthcare professionals may find it challenging, they must overcome the barriers to involving patients and relatives [70]. The present study highlights the need to further develop healthcare professionals’ competence in this area and future studies are required in order to learn more about what issues patients and relatives perceive as important and their views on the best way of being involved.

Involvement presupposes information. According to Norwegian law [71, 72] and The guide for next of kin [73], healthcare professionals need the patient’s consent before giving information about her/his health and care to relatives. However, this should not hinder nurses and physicians from communicating with relatives in a general way about treatment and care, their own situation and their support needs. For instance, both patients [74] and relatives [75] have reported that they prefer information about the expected course of the illness. However, healthcare professionals may feel uncomfortable about discussing end-of-life issues with patients and relatives because of feeling unprepared as well as worry about destroying hope [74, 76]. Nevertheless, in order to meet relatives’ information needs, healthcare professionals should investigate what they can tell them about the patient’s situation and what patients and relatives want to know. Lack of information has been found to cause frustration, powerlessness and loss of control [77, 78].

Professional communication about end-of-life questions assumes highly competent and sensitive professionals. In general, this is challenging in Norwegian community healthcare, where competence is relatively low [79]. However, in the present study, patients and relatives perceived that they were fairly secure concerning the professionals’ competence. This might be due to the organization, which aims to staff the SDWTs with healthcare professionals who are competent in palliative care. Nevertheless, there are variations and we know that adequate competence is not always available at night, or on weekends and holidays. It is necessary to bring palliative competence to those who need it in urban and rural areas 24/7. Due to scarce resources in Norwegian community care, it is essential to consider increased use of tele-health [80], not as a substitute for, but as a complement to healthcare professionals.

Patients rated one dimension and one aspect higher on the SI- than on the PR scale, whereas relatives rated all dimensions and aspects higher. This is in accordance with another study about quality of care among dyads of patients with lung cancer-family members, which found that family members generally rated the importance of the care higher than the patients and the SI higher than the PR [27]. A common understanding of patient-relative care preferences can facilitate adaption to the situation, whereas diverging perceptions can lead to conflicts. For instance, it is reported that relatives may diverge from patients in their preference for homecare services; patients may prefer to stay at home without help [81], whereas relatives expect that homecare services may ease their responsibilities as caregivers [13]. Knowing what is important to patients and relatives is essential for guiding politicians and leaders in planning and prioritizing future palliative homecare, as well as for enabling healthcare professionals to provide palliative care in accordance with decisions and promises. More studies are needed about these issues with a larger sample of patient-relative dyads.

One intention of SDWTs is to ensure continuity. Therefore, it was surprising to find that both patients and relatives scored the aspect of continuity in the care received as “fairly insecure” (PR scale) and of “great importance” on the SI scale. Another study has shown that patients and their families perceived lack of continuity as problematic because unknown healthcare professionals who were unfamiliar with their needs made the care unpredictable [51]. Other studies have found that visits by different healthcare professionals are relatively unproblematic. They report that the most important aspect for patients and their families is that the care is perceived as secure and of high quality [22, 82], which presupposes competent and available palliative homecare teams 24/7 and that they know the healthcare professionals involved [20, 82]. We suggest that future research should explore which palliative homecare models are appropriate for ensuring easily accessible and competent assistance.

Another aim of SDWTs is to assign each patient a primary contact. The primary contact is intended to contribute to continuity of care by being well informed and responsible for updating the documentation [82, 83], which has been found to be appreciated by patients [17] and family members [51]. It was therefore surprising that many patients and relatives in the present study reported that they lacked a primary contact and did not know how to obtain help if something unanticipated should happen. This underlines the importance of leaders ensuring that practice is in accordance with decisions and policy.

### Methodological considerations

The number of participants in the present study is low. However, they represent dyads in a vulnerable group and as such, the sample is not that small. Although patients with different diagnoses are represented, they are not fully representative of the population of patients in the late palliative phase. Furthermore, the sample is from one municipality and the results cannot be generalised, but they can nevertheless provide managers and politicians with important information for future planning to improve the service. Other municipalities might also find the results informative and useful for developing the quality of palliative homecare.

Despite the fact that the first author (RH) was in regular contact with the SDWT leaders to remind them about the study and answer their questions about inclusion, it is possible that not all eligible patients were invited to participate. Therefore, the results should be treated with care. Nevertheless, the study may provide a valuable contribution to those who deliver and are responsible for developing palliative homecare.

At the time the study was designed (2013), few studies were found on the sense of security in palliative homecare comprising both patients and relatives and none that included both the care received and its subjective importance. We therefore chose to develop a modified version of the original QPP. Recently, other instruments have been designed to measure the quality of palliative care from the patient perspective [84] as well as the sense of security in palliative care among patients and their family members [85, 86]. The modified QPP developed in this study aimed to measure perceptions of security and contains the main areas from these instruments. It should be noted that it also adds the subjective importance scale to the instruments about security and basically rests on the theoretical foundation of the original QPP [39, 41].

The absence of knowledge about the psychometric properties of the modified version of the QPP used in this study calls for caution when interpreting the results. However, it should be noted that 21 out of 24 Cronbach alpha coefficients were 0.70 or higher. The mean scores presented in this study are slightly higher (visual inspection) than the results obtained in a previous study in palliative care settings in Norway using the QPP-palliative care instrument [46]. The differences may be explained by the organisation of homecare in palliative SDWTs in the present study, whereas in the other study personnel were not specifically allocated to caring for patients in the palliative phase, but cared for all patients in need of homecare.

Data collection methods may influence answers about perceptions of security in palliative care. While the relatives completed the questionnaire themselves, all patients had help filling it in. As the interviews took place in the home, the patients could find the most comfortable position and were told that they could take a break whenever they needed. The two researchers (BB, RH) who conducted the interviews and filled in the questionnaire were not employed in the municipality. They assured the patients that their answers would not influence their care. To ensure that the patient and relative answered from the same care period, the data collection was timed as closely as possible.

## Conclusions

This study shows both high and low values in patients’ perceptions of security in the palliative homecare received (PR), whereas there are no high scores from relatives. The patients scored the subjective importance of the care (SI) higher than PR in one dimension (ID) and one aspect (continuity), whereas relatives’ scores differed in all dimensions and aspects. When assessing the palliative care received, the study reveals that there was limited agreement within the patient-relative dyads on all three dimensions and on the aspect of coordination/cooperation. Studies about the sense of security in palliative homecare from the perspectives of patient-relative dyads are important as relatives often provide care and act as a spokesperson for the patient, but also because relatives themselves are in a stressful situation. What patients and relatives assess as important in palliative homecare can guide the further development of this area. The present study adds knowledge that can contribute to concrete aspects of security in palliative homecare. More studies with a larger sample are required to further develop the QPP Security instrument.

## Data Availability

The dataset generated and analysed for this study cannot be shared because the conditions of NSD and participants’ consent did not include sharing the dataset.

## Appendix

**Table 4:** Perceptions of the sense of security in palliative home care of patient and relative dyads; care received and subjective importance by dimensions and items, Context-specific scales with aspects and items

	Perceived reality (PR)		Subjective importance (SI)	
	Patients (*n* = 32)	Relatives (*n* = 32)	Patients (*n* = 32)	Relatives (*n* = 32)
	Mean (SD)	Mean (SD)	Mean(SD)	Mean (SD)
Dimensions and items				
Medical-technical competence				
I (the patient) receive(s) the best possible help for pain				
I (the patient) receive(s) the best possible help for symptoms				
I (the patient) receive(s) the best possible care				
I (the patient) receive(s) the best possible medical treatment				
I (the patient) receive(s) correct doses of medication				
I (the patient) receive(s) medications in time				
I (the patient) receive(s) the best possible help with personal hygiene				
Physical – technical competence				
I have (the patient has) access to necessary equipment				
Identity-oriented approach				
I have (the patient has) a good opportunity to participate in decisions that apply to my (the patient’s) care				
I have (the patient has) a good opportunity to participate in decisions that apply to my (the patient’s) medical treatment				
I have (the patient has) a good opportunity to participate in (the patient’s) decisions that apply to the place where I (the patient) receive(s) treatment and care				
I (the patient) receive(s) useful information on my (the patient’s) illness and symptoms				
I (the patient) receive(s) useful information about what I (the patient) may expect of my (the patient’s) illness. and functional ability				
I (the patient) receive(s) useful information on how tests and treatment will take place				
I (the patient) receive(s) useful information on tests and treatment results				
I (the patient) receive(s) useful information on how I (the patient’s) should take medication				
I (the patient) receive(s) useful information on which physician is responsible for my (his/her) medical care				
I (the patient) receive(s) useful information on which RN is responsible for my (his/her) care				
I (the patient) get(s) honest answers to my (his/her) questions				
I am (the patient is) met with respect by				
- Carers				
- Physician				
I am (the patient is) welcome to talk to the personnel about what is important for me (him/her)				
I (the patient) experience(s) that the personnel understand how I (he/she) feels				
- Carers				
- Physicians				
I (the patient) experience(s) that the personnel show me (him/her) thoughtfulness				
- Carers				
- Physicians				
Context-specific scales; aspects and items				
Aspect: Competence				
The nurses’ knowledge is sufficient to give me (the patient) good care				
The physician’s knowledge is sufficient to give me (the patient) good medical treatment				
The knowledge of other personnel is sufficient to give me (the patient) good care				
The personnel know how to carry out procedures				
Aspect: Continuity				
I (the patient) experience(s) mostly being cared for by the same carer)				
I (the patient) experience(s) knowing in advance which carer is coming to me (the patient)				
I (the patient) experience(s) knowing at what time the carer will come				
I (the patient) experience(s) mostly receiving medical treatment from the same physician				
Aspect: Coordination and cooperation				
My (the patient’s) medical treatment from different physicians is well coordinated				
The care is well coordinated				
The care and treatment between carers and physicians is well coordinated				
The cooperation between home care and institutions is good				
Aspect: Availability				
I have (the patient has) access to qualified help when needed				
I have (the patient has) access to an institution when needed				
I have (the patient has) access to care promised by carers				
I have (the patient has) access to medical treatment promised by physicians				
I (the patient) experience(s) that the personnel/staff have time for me (him/her)				
- Carers				
- Physicians				

Scale PR:1 = Totally insecure, 2 = Fairly insecure, 3 = Fairly secure, 4 = Totally secure

Scale SI: 1 = Of no importance, 2 = Of some importance, 3 = Of great importance, 4 = Of very great importance

## Abbreviations

C-aspects: Contextual aspects
ID: Identity-orientation
MT: Medical – technical competence
PR: Perceived reality
PT: Physical-technical conditions
QPP: Quality from the Patient’s Perspective Instrument
RN(s): Registered nurse (s)
SC: Socio-cultural atmosphere of the care organisation
SDWT: Self-directed work teams
SI: Subjective importance
